# Immediate Insertion of an Artificial Lower Jaw after Removal of the Natural One

**Published:** 1889-04

**Authors:** 


ARTICLE VI.

### IMMEDIATE INSERTION OF AN ARTIFICIAL LOWER JAW AFTER REMOVAL OF THE NATURAL ONE.

A very interesting case is published in the November number *L'Odontologie* of the immediate insertion of a new lower jaw after removal of the old one for necrosis. The patient was a young man, age 21, who was admitted into the hospital at Lyons on 3rd December, 1878, and whose lower jaw, after six years of trouble from a tender tooth—evidently a pulpless one—had become necrosed almost in its entirety. Numerous sinuses formed both inside and outside the mouth, some of them being in the neck—a short distance from the clavicle. The patient acquired mucous rales all over the extent of the lungs, his cough became worse and worse, and he was hourly expected to begin to show symptoms of purulent infection, as the pus, which was poured out in great quantity, was almost all swallowed.

Letievant operated on the 14th December, 1878. This presented no great difficulties. All the body of the bone was lifted out with forceps, leaving the periosteum behind attached to the muscular tissue around. The periosteum had to be artificially stripped off from the ascending rami. On the left side all that was left of the lower jaw bone was the condyle and the coronoid process connected together by a portion of the ascending ramus; on the right side all that was left was the condyle cut off at the level of its neck. After the wound was cleansed, M. Claude Martin inserted into the periosteal gutter a counterfeit lower jaw made of rubber. He had previously taken an impression of the mouth, and made the rubber jaw in its entirety, and simply had to trim it down at the time of the operation.

The lower jaw was attached to an upper palate plate by spiral springs, with swivels, in the ordinary way, and the springs were made sufficiently strong to slightly tend to open the mouth, as the mouth could be closed to some slight extent by muscular action, but, there was nothing to help open the mouth but the springs. In the middle line the rubber jaw was divided with a saw, and the two halves held together by a spring controlled by a button, which allowed of its more easy introduction into the mouth. The fixing of the apparatus only took five minutes. The results of the operation were simple. The apparatus was well borne. After the first day after the operation, the patient ate better than with his diseased jaw. Excepting for a bad attack of erysipelas which came on five days later, and spread over the whole face and hairy scalp, and retarded progress for some time, the patient made an uninterrupted recovery. New bone was laid down from the periosteum beneath and around the rubber jaw; the various movements of the jaw re-established themselves. The rubber jaw was reduced from time to time to allow for deposit of new bone. A new apparatus was at length made to better adapt itself to the new bone; it was made intentionally heavy, and the springs which united it to the palatine plate were set farther forward than usual in order to obtain a propulsion of the lower jaw, forwards and downwards, and to direct the ossification of the chin in those directions. A third was finally made smaller than any of the preceding ones and without springs. The operator remarks that the apparatus might be compared to Meckels' Cartilage, which directs the process of ossification, and in so far as it effects this, its function diminishes, until it disappears altogether as soon as the new bony jaw can go alone in safety.

At the time of the patient leaving the hospital, ordinary sensibility of the skin of the chin had completely returned, whereas it had been for some time very much blunted.

Ten years after the removal of the original jaw there was no contraction of the upper jaw observable.

This gratifying result was attributable to the mechanical action of the artificial jaw, in keeping the parts in a normal state of extension during the healing process, as in the absence of such help there is deformity, caused by contraction after excision of the lower jaw, though in this connection Bryant remarks:—"The amount of regeneration of bone will depend greatly on the state of the periosteum during the removal of the sequestra. If this be healthy, and if new bone have already formed prior to operation, a very perfect reproduction of the portions of jaw removed may take place; in fact, complete reproduction of the whole lower jaw, body, rami, and epiphyses, though in a somewhat rudimentary and imperfect form, may follow its removal for phosphorous necrosis. . . . Should no new bone have formed before the operation, a dense fibroid cicatrical structure will replace the lost bone;" and, again, in another place he says, "When cicatrization is complete, a dense mass of fibrous tissue is formed in the place of the jaw, and comparative little deformity results." But here it is not quite clear whether he means after a total or only a partial excision.—*Londou Dental Record.*

